# Bone marrow T cells from the femur are similar to iliac crest derived cells in old age and represent a useful tool for studying the aged immune system

**DOI:** 10.1186/1742-4933-10-17

**Published:** 2013-05-04

**Authors:** Theresa Pritz, Katja Landgraf-Rauf, Dietmar Herndler-Brandstetter, Rauend Rauf, Julian Lair, Robert Gassner, Birgit Weinberger, Martin Krismer, Beatrix Grubeck-Loebenstein

**Affiliations:** 1 Institute for Biomedical Aging Research, University Innsbruck, Rennweg 10, Innsbruck, Austria; 2 Department of Orthopedic Surgery, Innsbruck Medical University, Anichstrasse 35, Innsbruck, Austria; 3 Department of Cranio-Maxillofacial and Oral Surgery, Innsbruck Medical University, Anichstrasse 35, Innsbruck, Austria; 4 Present address: Yale University School of Medicine, 333 Cedar Street, New Haven, CT, USA

**Keywords:** Bone marrow, Iliac crest, Femur, Peripheral blood, Mononuclear cells, T cells, Cytokines, T cell lines, Cytomegalovirus

## Abstract

**Background:**

CD4^+^ and CD8^+^ T cells reside in the human bone marrow (BM) and show a heightened activation state. However, only small sample sizes are available from sources such as the iliac crest. Larger samples can be obtained from the femur in the course of hip replacement surgery. It was therefore the goal of the present study to compare the phenotype and function of BM T cells from different sources from elderly persons and to investigate how femur derived bone marrow T cells can serve as a tool to gain a better understanding of the role of adaptive immune cells in the BM in old age.

**Results:**

Bone marrow mononuclear cells (BMMC) were isolated from either the iliac crest or the femur shaft. As expected the yield of mononuclear cells was higher from femur than from iliac crest samples. There were no phenotypic differences between BMMC from the two sources. Compared to PBMC, both BM sample types contained fewer naïve and more antigen experienced CD4^+^ as well as CD8^+^ T cells, which, in contrast to peripheral cells, expressed CD69. Cytokine production was also similar in T cells from both BM types. Larger sample sizes allowed the generation of T cell lines from femur derived bone marrow using non-specific as well as specific stimulation. The phenotype of T cell lines generated by stimulation with OKT-3 and IL-2 for two weeks was very similar to the one of *ex vivo* BM derived T cells. Such lines can be used for studies on the interaction of different types of BM cells as shown by co-culture experiments with BM derived stromal cells. Using CMV_NLV_ specific T cell lines we additionally demonstrated that BM samples from the femur are suitable for the generation of antigen specific T cell lines, which can be used in studies on the clonal composition of antigen specific BM T cells.

**Conclusion:**

In conclusion, our results demonstrate that BMMC from the femur shaft are a useful tool for studies on the role of T cells in the BM in old age.

## Background

Recent studies indicate that the bone marrow (BM) is a lymphoid organ which is important for adaptive immune responses [1]. In mice it has been shown that a major proportion of memory CD4^+^ and CD8^+^ T cells is located in the BM and remains there for a prolonged period of time in distinct survival niches [2]. The situation seems to be similar in humans [3].

Yet, little information is available on the role of the human BM in adaptive immune responses during aging. Our group has contributed to this topic using BM from iliac crest from young and elderly persons. Our results show that the number of CD4^+^ and CD8^+^ T cells in the BM is maintained during aging and that these cells are in a heightened activation state. However, the composition of the T cell pool in the aged BM is altered with a decline of naïve and an increase in effector memory T cells [4].

In previous studies we used iliac crest samples from patients undergoing reconstructive surgery and bone remodeling of the jaw following accidents or for dental implants. The major advantage of this source of bone marrow is that it is obtained from healthy donors of different age groups. However, the samples are generally very small with a low yield of BMMC. For many research questions higher cell numbers are needed. BM samples obtained from healthy donors by aspiration, e.g. for bone marrow transplantation frequently contain large numbers of peripheral mononuclear cells [5], which makes them unsuitable for studies in which e.g. T cell composition in the bone marrow is analyzed in direct comparison to peripheral blood. We therefore tested a different source of BM, namely the femur shaft. BM from the femur shaft is available from patients undergoing total hip replacement [6]. Following collagenase digestions it is possible to isolate relatively high numbers of BMMC from these samples. A potential problem may be the close vicinity of the femur shaft to the inflamed joint in patients who undergo hip replacement, or the fact that these patients are frequently treated with anti-inflammatory drugs. In order to be suitable for studies on the physiology of the aging immune system the possibility that inflammatory processes and/or drugs could influence the composition and activation state of adaptive immune cells needs to be excluded. It was therefore the goal of our present study to compare the phenotype and function of BM T cells from the iliac crest and the femur shaft. We show that inflammatory processes in the joint do not affect the phenotype and function of T cells in the BM obtained from femur samples. We also suggest approaches how BMMC from the femur can be used for studies on the role of BM T cells in old age.

## Results

### Comparison of CD4^+^ and CD8^+^ T cell phenotypes within BM from different sources

**Figure 1:**
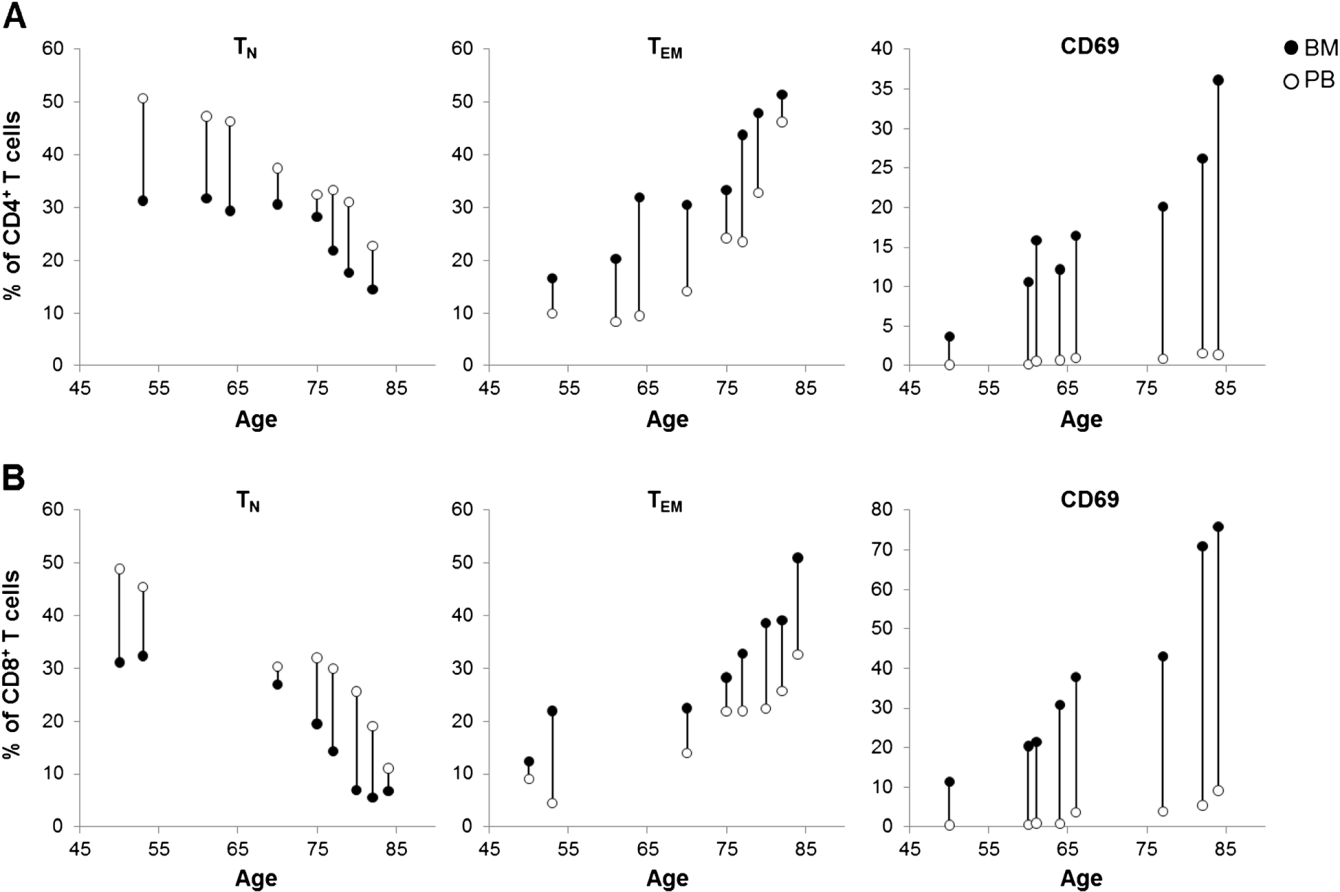
**The impact of aging on CD4**^**+ **^**and CD8**^**+ **^**T cells in the BM. ***A*, CD4^+^ T cells: the graphs show the impact of aging on the different subsets. Naïve (T_N_, CD45RA^+^CCR7^+^), effector memory (T_EM_, CD45RA^-^CCR7^-^) as well as CD69 expressing T cells in BM from the femur (black dots, n = 8) in comparison to autologous blood samples (white dots, n = 8) are shown. *B*, CD8^+^ T cells: the graphs show the impact of aging on the different subsets. Naïve (T_N_, CD45RA^+^CCR7^+^), effector memory (T_EM_, CD45RA^-^CCR7^-^) as well as CD69 expressing T cells in BM from the femur (black dots, n = 8) in comparison to autologous blood samples (white dots, n = 8) are shown. CD4 and CD8 staining were performed on samples from different donors.

We first compared the composition of the CD4^+^ and CD8^+^ T cell pool in BM obtained from either the femur or iliac crest from elderly persons. Expectedly, the yield of BMMC was higher from femur than from iliac crest samples (21.6x10^6^ ± 2.5; n = 13: femur versus 4.77 × 10^6^ ± 1.3; n = 13: iliac crest; p < 0.001). We analyzed the expression of the surface molecules CD45RA and CCR7 to define naïve (CD45RA^+^CCR7^+^), central memory (CD45RA^-^CCR7^+^), effector memory (CD45RA^-^CCR7^-^) and T_EMRA_ cells (CD45RA^+^CCR7^-^). The relative numbers of CD4^+^ as well as CD8^+^ T cell subsets were similar in samples obtained from the femur and the iliac crest (Table 1). We were able to confirm characteristic differences between BMMC and PBMC, which were previously described by our group [3,4]. While the numbers of naïve CD4^+^ T as well as CD8^+^ T cells were decreased in both types of BM compared to PB, there was an increase in the numbers of effector memory T cells in the BM. In accordance with our previous results, a relatively large percentage of BM T cells expressed CD69. Again there were no differences in the number of CD69^+^ cells in BM samples from femur and iliac crest. In addition to the data in Table 1, we plotted the data on T cell subsets versus age as a continuous variable for femur BM and PB, and linked BMMC and PBMC values in each donor (Figure 1). Characteristic age related changes in the relative numbers of T_N_ and T_EM_ can be seen. The impact of CMV infection on the distribution of T cell subsets in the BM from iliac crest as well as from femur is similar to what is known from the periphery ([4] and data not shown).

**Table 1:** Comparison of mononuclear cells from different sources of BM and from peripheral blood (PB)

	**BM**			**PB**
**CD4**^**+**^	**Femur**	**Iliac crest**	**P**^**#**^	
	**(n = 8)**	**(n = 8)**		**(n = 16)**
% CCR7^+^CD45RA^+^ (T_N_)	26.1 ± 2.3	19.8 ± 1.9	0.08	34.8 ± 2.9^*/§^
%CCR7^+^CD45RA^-^ (T_CM_)	21.9 ± 5.9	24.4 ± 3.1	0.89	28.8 ± 3.7
% CCR7^-^CD45RA^-^ (T_EM_)	34.6 ± 4.4	44.3 ± 3.6	0.11	23.8 ± 3.3^*/§^
%CCR7^-^CD45RA^+^ (T_EMRA_)	12.1 ± 3.3	13.4 ± 3.0	0.80	15.7 ± 2.7
CD69	16.9 ± 3.1	15.9 ± 2.3	0.69	0.7 ± 0.1^*/§^
**CD8**^**+**^	**Femur**	**Iliac crest**		
	**(n = 8)**	**(n = 8)**		**(n = 16)**
% CCR7^+^CD45RA^+^ (T_N_)	17.8 ± 3.5	14.1 ± 3.2	0.47	29.3 ± 3.4^*/§^
%CCR7^+^CD45RA^-^ (T_CM_)	16.7 ± 3.1	10.9 ± 4.1	0.68	14.5 ± 2.2
% CCR7^-^CD45RA^-^ (T_EM_)	29.1 ± 4.2	34.1 ± 6.7	0.64	21.9 ± 2.6^*/§^
%CCR7^-^CD45RA^+^ (T_EMRA_)	33.0 ± 0.5	41.6 ± 5.9	0.59	39.5 ± 3.7
CD69	39.0 ± 6.1	46.3 ± 4.1	0.59	1.9 ± 0.4^*/§^

BM was obtained either from the femur shaft or from iliac crest, as described in Materials and Methods. All samples were from elderly persons (mean age ± SEM: femur 68.6 ± 2.1 years; iliac crest 66 ± 3.1 years). Data were calculated using *t* test and are presented as mean ± SEM. ^*^p < 0.05: PBMC vs. femur derived BMMC; ^§^p < 0.05: PBMC vs. iliac crest-derived BMMC. There were no significant differences between samples obtained from the two different sources of BM. ^#^P values: femur versus iliac crest.

### BMMC obtained from the femur and iliac crest have a similar cytokine production pattern

**Figure 2:**
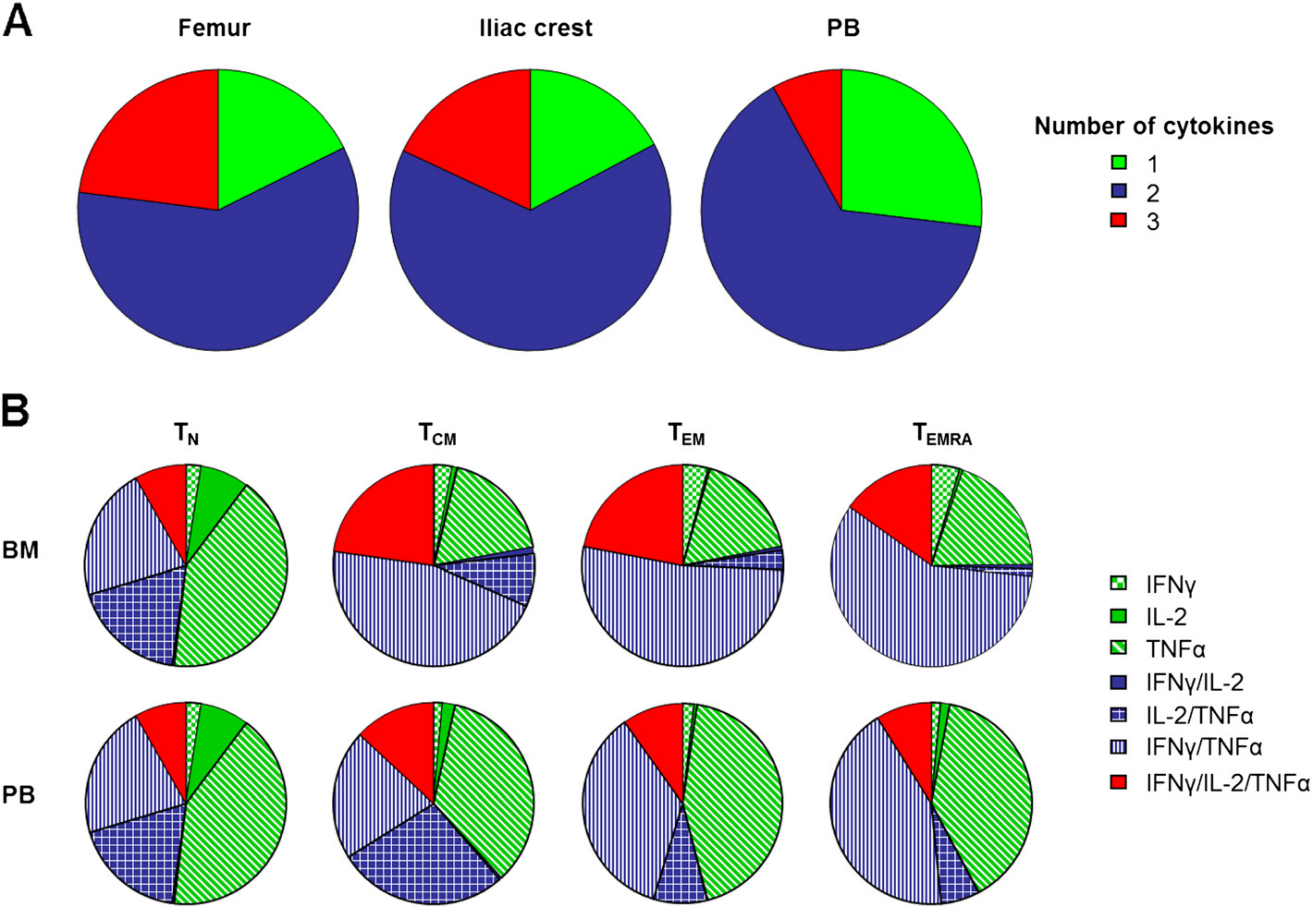
**Cytokine production by CD8**^**+ **^**T cell in the BM and the PB.** BMMC from the femur (n = 14) or iliac crest (n = 5) and PBMC (n = 19) from healthy elderly persons were stimulated with PMA and ionomycin in the presence of brefeldin A for 4 h and the production of the cytokines IFN-γ, TNF-α and IL-2 was determined by intracellular FACS analysis. *A*, CD8^+^ T cells producing cytokines after stimulation are depicted and the pie charts show the percentages of CD8^+^ T cells producing one (green), two (blue) or three cytokines (red). B, Cytokine production of CD8^+^ naive (T_N_), central memory (T_CM_), effector memory (T_EM_), and T_EMRA_ was analyzed in femur derived BMMC (n = 12) and PBMC (n = 12) from the same donors. The pie chart represents percentages of T cells expressing different combinations of cytokines as indicated.

Intracellular cytokine staining was performed in order to assess possible differences in cytokine production in BMMC isolated from the femur or the iliac crest. In a first set of experiments the occurrence of polyfunctional T cells was analyzed within the total CD4^+^ and CD8^+^ T cell populations. The occurrence of polyfunctional T cells was similar in cells derived from either the femur or the iliac crest. In accordance with our previous results [3,4], the number of cells secreting three cytokines was higher in T cells from the BM than from the PB (p < 0.05). Data on total CD8^+^ T cells are depicted in Figure 2A. Similar results were obtained for CD4^+^ T cells (data not shown). As higher numbers of BMMC were available from the femur than from the iliac crest, a more detailed analysis of cytokine production was possible using femur derived cells. The cytokine production profile of naïve, central memory, effector memory and T_EMRA_ cells isolated from femur derived BM was compared to the cytokine production in corresponding subsets from the PB. Cytokine production by CD8^+^ T cell subsets is shown in Figure 2B. Following stimulation, the majority of naïve T cells from both sources produced only one cytokine, mostly IL-2 or TNFα. The remaining cells produced either a combination of IL-2 and TNFα or of IFNγ and TNFα, while the fraction of triple positive cells was relatively small in both types of naïve samples. In contrast, the percentage of cells producing only one cytokine was significantly lower in antigen experienced cells derived from the BM than in corresponding populations from the PB (p < 0.05). In accordance with this, the percentage of double/triple positive cells was higher in antigen experienced cells from the BM than in the corresponding subpopulations from the PB (p < 0.05). Expectedly, the percentage of IL-2 producing cells decreased with T cell differentiation, while the percentage of IFNγ/TNFα producing cells was highest in the T_EMRA_ subsets and even further increased in the T_EMRA_ population from the BM compared to the PB (p < 0.05). Similar results were obtained for CD4^+^ T cells (data not shown). Our results demonstrate that T cells from the BM are functionally more active than corresponding cells from the PB and that BM derived T cells of a high differentiation stage can produce a variety of different cytokines.

### Generation of T cell lines from BM samples by non-specific stimulation

For many functional tests, in particular studies on the interaction of T cells with other cell types such as stromal cells, the number of *ex vivo* obtainable T cells is not high enough, even when BM samples from the femur are used. We therefore expanded BM derived T cells from femur samples from two donors by stimulation with an anti-CD3 antibody and IL-2. After two weeks of culture we were able to increase the number of T cells by approximately 20-fold. We then analyzed the phenotype of the obtained lines in comparison to the original *ex vivo* populations. In accordance with our previous findings the BM samples contained more CD8^+^ than CD4^+^ T cells, which is in contrast to the situation in peripheral blood [3,4]. After two weeks in culture the ratio of CD4^+^/CD8^+^ T cells was further decreased. However, the percentage of CD28^-^ T cells was relatively constant during the cultivation period and corresponded to the population obtained *ex vivo* (data not shown). Non-specifically expanded T cell lines are therefore a suitable model to study the function of BM derived T cells.

### Effect of BM derived stromal cells on bone marrow derived T cell lines

**Figure 3:**
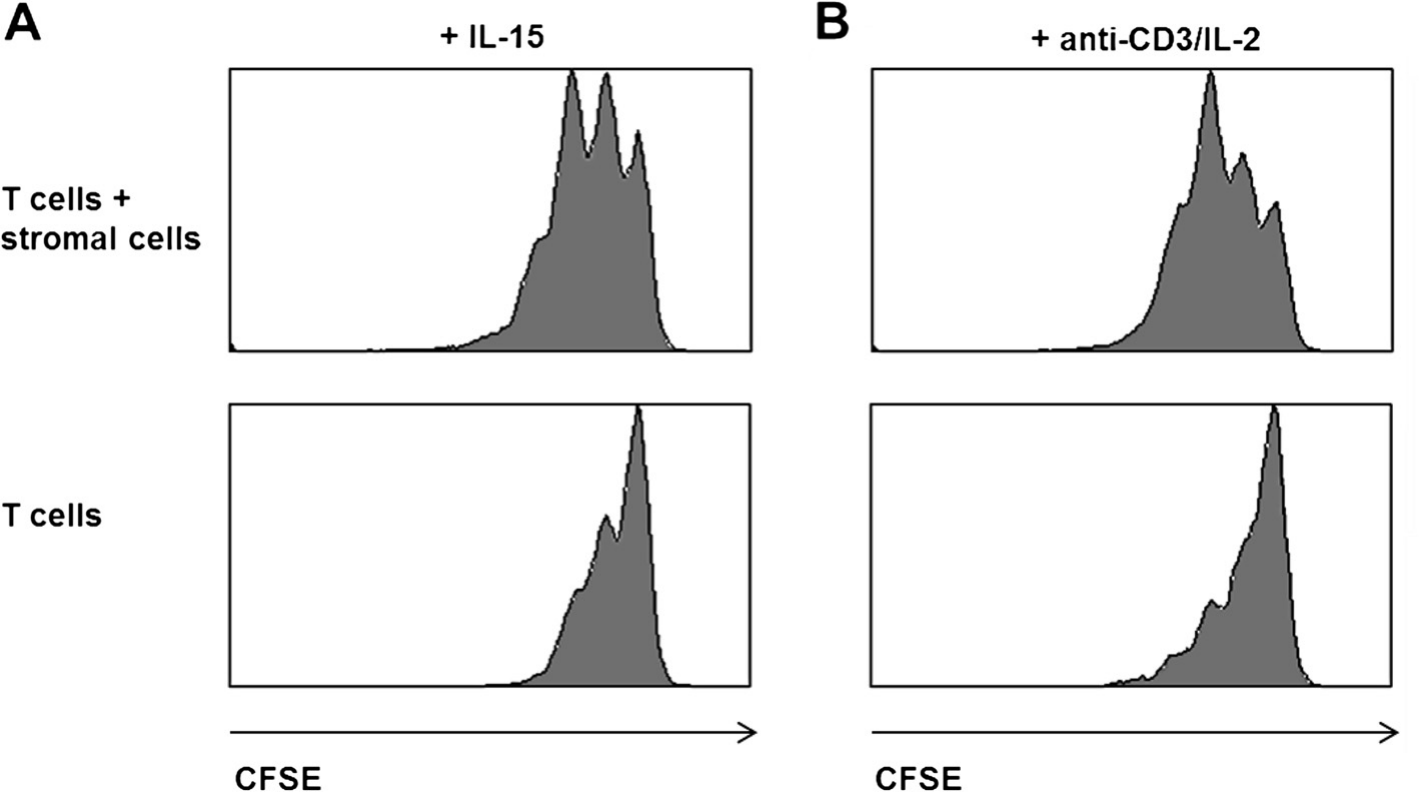
**Proliferation of T cells in the presence or absence of stromal cells.** T cell lines generated from BMMC from the femur as described in Materials and Methods were stimulated with IL-15 (50 ng/ml) or anti-CD3 (3 ng/ml) and IL-2 (20 ng/ml) for four days in the presence or absence of stromal cells. T cells were labeled with CFSE and proliferation was measured on day 5. One representative example of two independent experiments is shown.

In addition to lymphocytes, stromal cells can be isolated from human BM. These stromal cells contain mesenchymal stem cells, which can be isolated and cultivated *in vitro*[7] and have suppressive effects on T cell function [8]. However, the effect of other stromal cell types on T cell function is less well studied and we therefore tested the suitability of BM derived T cell lines for studies on the interaction of T cells and stromal cells. BM derived T cell lines were stimulated with either IL-15 (homeostatic stimulation) or a combination of anti-CD3 antibody and IL-2 (antigenic stimulation) in the absence or presence of autologous stromal cells. The presence of stromal cells had no influence on the T cell phenotype (data not shown). Our results additionally showed that BM stromal cells did not have a suppressive effect on the proliferation of BM derived T cell lines in response to stimulation (Figure 3).

### The clonal composition of the CD8^+^ T cell pool in the BM in comparison to the PB: The example of CMV specific cells

**Figure 4:**
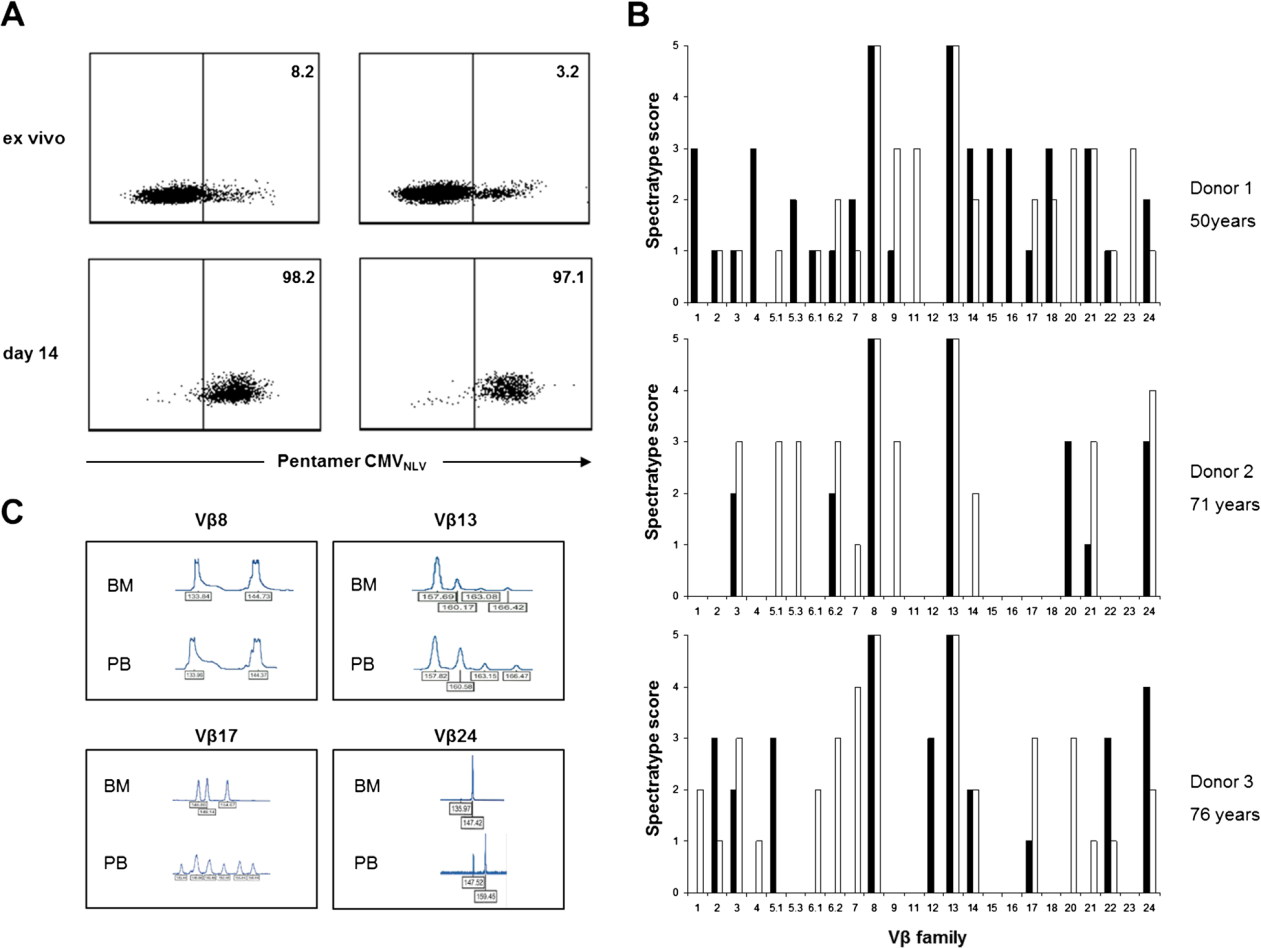
**Analysis of CMV**_**NLV **_**specific T cells *ex vivo *and after 14 days in culture.** PBMC as well as femur derived BMMC from HLA-A2 positive donors were cultivated in the presence of CMV_NLV_ peptide (0.1 μg/m) and IL-2 (20 ng/ml) for two weeks. *A*, T cells were stained with PerCP-conjugated anti-CD8 antibody and APC-conjugated pentamers containing the CMV_NLV_ peptide. Gating on CD8^+^ T cells was performed. A representative example is shown for CMV_NLV_ peptide specific CD8^+^ T cells from the BM (left panel) and PB (right panel) before culture as well as after two weeks of culture with the peptide CMV_NLV_ and IL-2 followed by the purification of the CMV_NLV_ specific population with magnetic beads as described in Materials and Methods. The staining depicts one of three similar experiments. *B*, Spectratyping of the CDR3 region was performed on purified CMV_NLV_ specific T cells derived from BM and PB. Representative results comparing the clonal composition of CMV_NLV_ specific T cells derived from BM and PB for selected Vβ families are shown. The relative size of the CDR3-region fragments is indicated below the peaks. *C*, Clonality and intensity scores were determined for spectratyping results of 24 individual Vβ families for CMV_NLV_ specific T cells derived from BM (black) and PB (white). Results are depicted for three individual donors. The clonality score (1 = Gaussian distribution; 2 = several peaks; 3 = one peak) and intensity score (0 = <500 RFU [relative fluorescence units]; 1 = 500-3000 RFU; 2 = 3000-8000 RFU; 3= >8000 RFU) were added.

To analyze the clonal composition of BM derived T cells of a certain specificity, we stimulated BMMC as well as PBMC with the peptide CMV_NLV_ for two weeks following a previously published protocol [9,10]. It was of interest that the starting population from the BM contained more CMV_NLV_ specific cells than the corresponding population from the PB (12.5% ± 3.2, n = 5: BM versus 5.0% ± 3.6; n = 5: PB; p < 0.01). A representative staining is shown in Figure 4A. BMMC and PBMC from three donors were used for the establishment of antigen specific T cell lines. After 14 days of culture CMV_NLV_ specific T cells were purified using allophycocyanin (APC)-conjugated CMV_NLV_ pentamers, anti-APC-antibodies coupled with magnetic beads and MACS technology. The purity of the 6 T cell lines was above 95% after purification (Figure 4A). RNA was isolated from the T cell lines and cDNA synthesis was performed using a reverse transcription system, as described in Materials and Methods. TCR fragments were amplified from cDNA for 24 Vβ families (BV) and complementarity determining region (CDR3) spectratyping was performed. While some cDNA clones, in particular within the Vβ 8 and Vβ 13 family, were identical in the T cell lines derived from the BM and the PB (Figure 4B, upper panel), other cDNA clones occurred exclusively in the BM or in the PB (Figure 4B, lower panel). This fact is further underlined by the results depicted in Figure 4C, in which the spectratyping data of the 6 CMV_NLV_ specific T cell lines obtained from three individual donors are summarized. The CMV_NLV_ specific T cell lines generated from donor 1 were both relatively polyclonal, which was most likely due to the fact that this donor was younger than the other two donors from whom we obtained material (donors 2 and 3). There were still 5 Vβ families (1, 4, 5.3, 15 and 16), in which cDNA clones were only found in the BM derived, but not in the peripheral T cell line. On the other hand, 4 Vβ families (5.1, 11, 20 and 23) only contained cDNA clones in the peripheral, but not the BM derived CMV_NLV_ specific T cell line. In comparison, the T cell lines generated from donors 2 and 3 had a more restricted diversity and this restriction seemed to be even more pronounced in BM derived CMV_NLV_ specific T cell lines. Thus, Vβ 20 positive cDNA clones were only detected in the BM derived T cell line, but not in the peripheral line, Vβ 5.1, 5.3, 7, 9 and 14 cDNA clones were only found in the peripheral T cell line, but not in the BM derived one from donor 2. The picture was similar in the CMV_NLV_ specific T cell lines derived from donor 3: Two Vβ families (5.1, 12) contained CMV_NLV_ specific cDNA clones in the BM derived line only, while 7 Vβ families (1, 4, 6.1, 6.2, 7, 20, 21) contained cDNA clones in the peripheral, but not in the BM derived T cell line.

## Discussion

In the present study we used femur as well as iliac crest to isolate BMMC from the BM of elderly persons and compared the phenotype and function of T cells within these two preparations. In the past we used iliac crest samples from patients undergoing reconstructive surgery and bone remodeling of the jaw following accidents or for dental implants. These persons are known to be healthy and are not treated with any immunemodulatory drugs. In contrast, femur BM is obtained from patients undergoing hip replacement surgery. They suffer from inflammation of the joint and are frequently treated with anti-inflammatory drugs. Both factors might affect the composition of the T cell pool as well as the activation state of BM derived T cells from the femur shaft region, which could compromise the suitability of this material for functional analyses of BM derived T cells. We clearly demonstrate that this is not the case, as the size of the different T cell subsets, their activation state, and their cytokine production profile was not different between samples obtained from the two tissue sources. We can therefore conclude that BM from the femur shaft can be considered as healthy and suitable for physiological studies.

As the yield of BMMC from the femur is significantly larger than from the iliac crest, femur BMMC can be used for more sophisticated experimental approaches leading to more detailed results. In samples from the iliac crest it was only possible to define the cytokine production of the total CD8^+^ and CD4^+^ T cell populations. However, using femur derived BMMC we were able to analyze the production of several cytokines separately in T cell subsets, namely naïve, central memory, effector memory and T_EMRA_ cells. These data were of interest, as T cells of late differentiation stages were more frequently polyfunctional in the BM than in the PB. These data support our concept that CCR7^-^ cells, which are otherwise susceptible to apoptosis-inducing stimuli and destined to perish [11], may not only survive, but also function in the IL-15 rich BM microenvironment in old age [3,4]. Whether these highly differentiated T cells in the BM of elderly people represent a useful line of defense in the absence of fully functioning naïve T cells [12,13] or take up space otherwise reserved for CD4^+^ T cells of an earlier differentiation stage or for long-lived plasma cells [14,15] is not yet known. Further studies will be needed to clarify this open question.

One possible approach to address these issues is the *in vitro* modeling of the *in vivo* situation by co-culturing BM derived T cells and BM derived stromal cells. As in humans both cell types are not available in sufficiently high numbers for functional analysis in *ex vivo* samples, even in the relatively large BM samples from the femur, the generation of cell lines is a prerequisite to allow conclusive experimental setups. Our results show that T cell lines generated from BMMC by non-specific stimulation maintain the characteristic phenotype of the original population. They also demonstrate that proliferation of the lines in response to stimulation with both, anti-CD3 antibody or IL-15, is not negatively affected by the presence of stromal cells. This may be surprising, as there are multiple reports in the literature that BM derived mesenchymal stem cells have immunsuppressive effects on T cells [16]. Because of their immunomodulatory properties mesenchymal stromal cells are already being used in clinical trials for preventing graft vs. host disease and other disorders going along with increased immune responsiveness [17,18]. In view of our results it seems likely that only stromal cells of early differentiation stages, which may still be multipotent, are capable of immunosuppressive effects. Our *in vitro* model system will represent a useful tool to analyze how stromal cells of different phenotypes - from early differentiation stages to senescence - affect the function of different T cell subsets.

In this context it will also be important to analyze how stromal cells affect T cell responses to different antigens. Our data clearly indicate that the clonal composition of a CMV_NLV_ specific T cell population is different in the BM and in the periphery. With the exception of Vβ 8^+^ and Vβ 13^+^ T cells, which are known to dominate CMV_NLV_ specific responses [9,19], a relatively large number of clones is found in the BM, but not in the PB and vice versa. Our data also suggest that the CMV_NLV_ specific T cell repertoire in the BM is more restricted than in the periphery. It will be a challenge to identify BM specific clones, isolate them and analyze their avidity and function. A similar approach using peripheral T cells has in the past been successfully used by our group and others [20,21]. As CMV is believed to accelerate the aging of the immune system [22-24], it will be of special interest to define the exact role of CMV specific clones which reside in the BM in the infection / reactivation process.

## Conclusion

In conclusion, our results demonstrate that the BMMC population derived from the femur shaft provides a useful tool for a variety of immunological studies addressing questions of major importance. New insights thus obtained will improve our understanding of age-related changes of immune function in old age.

## Materials and methods

### Sample collection and preparation

Paired blood and BM samples were obtained from systemically healthy elderly persons. Individuals who suffered from diseases known to influence the immune system, including autoimmune diseases and cancer, were excluded from the study. Femur derived BM and autologous blood samples were obtained from a total of 32 donors (19 females, 13 males, mean age ± SEM: 68.6 ± 2.1 years). CMV specific antibodies were measured in serum. 24 donors were sero-positive and 8 donors were sero-negative for CMV. Iliac crest material and blood were obtained from a total of 30 donors (22 females, 8 males, mean age ± SEM: 66 ± 3.1 years). The CMV serostatus was only known from seven patients, 4 donors were CMV sero-positive and 3 donors were CMV sero-negative. Informed written consent was obtained and the study was approved by the Ethics Committee of Innsbruck Medical University. Bone from the femur shaft was harvested at the Department of Orthopedic Surgery at Innsbruck Medical University from patients undergoing hip replacement surgery. A biopsy of *substantia spongiosa ossium*, which would otherwise have been discarded, was used to isolate BMMC. BMMC samples from the iliac crest of elderly persons were obtained as previously described [3,4]. In brief, bone from the iliac crest was harvested at the Department of Cranio-Maxillofacial and Oral Surgery at Innsbruck Medical University for bone molding/recontouring prior to insertion into other areas of the body, in particular facial regions. A biopsy of *substantia spongiosa ossium*, which would otherwise have been discarded, was used to isolate BMMC. Results on some of the iliac crest derived samples used in this study have been described in a different context [3,4]. Both types of bone biopsies were washed once with complete RPMI 1640 (Lonza) supplemented with 10% FCS (Sigma Aldrich), 1% Penicillin/Streptomycin (PAA). Bone biopsies were then fragmented and treated with sterile-filtered, chromatographically purified collagenase (CLSPA; Worthington; 20 U/ml in complete RPMI) for 2 h at 37°C, 20% O_2_ and 5% CO_2_. Cells were centrifuged, and BMMC were purified by density-gradient centrifugation (Ficoll-Hypaque). Preparation of PBMC was also performed by density-gradient centrifugation.

### Flow cytometric analysis

Immunofluorescence surface staining was performed by adding a panel of directly conjugated mAb to freshly prepared PBMC, BMMC or T cell lines, respectively. The Abs used were: CD3 (PE-Cy7 and APC-Cy7), CD4 (FITC, PerCP and PE-Cy7), CD28 (APC), CD45RA (PE, PerCP and APC) CD69 (FITC and PE) (all BD Pharmingen), CD8 (PerCP; Biolegend) and CCR7 (FITC; R&D Systems). Cells were incubated with the antibodies for 30 min at 4°C. The labeled cells were measured by a FACSCanto II Instrument (BD Biosciences), and the data were analyzed using FACSDiva software (BD Biosciences).

### Intracellular cytokine staining

The production of cytokines by T cells from the BM and PB was assessed by stimulating the cells for 4 h with 30 ng/ml PMA and 500 ng/ml ionomycin in the presence of 10 μg/ml brefeldin A (all Sigma-Aldrich). Cells were permeabilized using the Cytofix/Cytoperm kit (BD Pharmingen) and intracellular staining of IFN-γ (FITC), TNF-α (PE) and IL-2 (PE-Cy7) (all BD Pharmingen) was performed following manufacturer´s instructions. In intracellular cytokine staining experiments naïve cells were defined as CD45RA^+^ CD28^+^, central memory cells as CD45RA^-^ CD28^+^, effector memory cells as CD45RA^-^ CD28^-^ and T_EMRA_ as CD45RA^+^ CD28^-^.

### Pentamer staining

For the detection of CMV pp65 NLVPMVATV (CMV_NLV_) peptide specific CD8^+^ T cells in HLA-A2 positive, CMV positive persons, the cells were stained with CMV_NLV_ Pro5® MHC Pentamer (Proimmune) and incubated for 10 min at RT in the dark before staining for other surface markers was performed.

### Cell culture

Cell culture experiments were performed with RPMI 1640 (Lonza) supplemented with 10% FCS (Sigma-Aldrich) and 1% Penicillin/Streptomycin (PAA).

For non-specific expansion, femur derived BMMC and PBMC from two HLA-A2 positive donors were stimulated with anti-CD3 (3 ng/ml) (BD Pharmingen) and IL-2 (20 ng/ml) (Novartis) for 2 weeks. During this time they were restimulated once a week with anti-CD3 antibody and every third day with IL-2.

To generate CMV_NLV_ peptide-specific CD8^+^ T cell populations, femur derived BMMC and PBMC from three HLA-A2 positive, CMV positive elderly donors were incubated with 0.1 μg/ml pp65 CMV_NLV_ peptide (NLVPMVATV; Bachem) for two weeks. During this time they were restimulated with IL-2 (20 ng/ml) (Novartis) every three days and with peptide once on day 7. After 14 days CMV_NLV_ specific CD8^+^ T cells were purified using MACS technology, as previously described [10]. Briefly, CMV specific CD8^+^ T cells were positively selected using APC-coupled CMV_NLV_ pentamers, followed by the addition of anti-APC-antibodies coupled with magnetic beads (all (BD Pharmingen) and a LS column (Miltenyi Biotec). The purity of the CMV_NLV_ specific CD8^+^ T cell lines was >95% after purification.

### TCR Vβ CDR3 spectratyping analysis

TCR Vβ transcripts of CMV_NLV_ specific T cells from the femur derived BM and PB were amplified by PCR using HotStarTaq Master Mix Kit (Qiagen), primers (MWG Biotech) specific for each of the 24 human Vβ families and a specific primer for the C region of the β-chain (labeled with the fluorescent dye marker 6-FAM) as previously described [19,25]. An aliquot of the PCR product was diluted in 20 μl deionized formamide and 1.2fmol internal lane standard GeneScan-350 Tamra (PerkinElmer). The samples were denatured at 90°C for 2 min and loaded on a CE 3100 Genetic Analyzer (PerkinElmer). Each sample was injected for 5 s at 15 kV and electrophoresed for 24 min at 10 kV using a 36-cm capillary and POP46 (PerkinElmer). Analysis of the raw data was performed applying the GeneScan 3.7 analysis software package (Applies Biosyststems) using the Local Southern method for fragment size estimation. For each Vβ family the occurrence of dominant clonal expansions was quantified by assigning scores for clonality and intensity as previously described [9]. The clonality score (1 = Gaussian distributed; 2 = several peaks; 3 = one peak) and intensity score (0 = <500 RFU [relative fluorescence units]; 1 = 500-3000 RFU; 2 = 3000-8000 RFU; 3= >8000 RFU) were added.

### Stromal cell culture

Following density gradient centrifugation the BMMC samples were incubated overnight at 37°C, 5%CO_2_. After this time stromal cells had adhered to plastic, the non-adherent T cell enriched population was removed and was used for analysis or further culture. The adherent cells were further incubated in RPMI 1640 (Lonza) supplemented with 10% FCS (Sigma-Aldrich) and 1% Penicillin/Streptomycin (PAA) for one to two weeks and then removed from the plastic by trypsin/EDTA (Sigma). They were a heterogeneous population consisting of morphologically defined classical stromal cells, monocytes, dendritic cells and fibroblasts.

### CFSE staining

To measure proliferation, T cells were stained with CFSE (Carboxyfluorescein succinimidyl ester). Briefly, cells were washed with PBS and stained with CFSE (Molecular Probes) for 10 min at 37°C. T cells were washed twice with RPMI 1640 (Lonza) supplemented with 10% FCS (Sigma-Aldrich) and 1% Penicillin/Streptomycin (PAA). Proliferation was measured after 5 days using a FACSCanto II Instrument (BD Biosciences), and the data were analyzed using FACSDiva software (BD Biosciences).

### Statistical anaysis

Differences between samples and groups were evaluated using paired or unpaired t tests, respectively. *p* values <0.05 were considered as statistically significant.

## Abbreviations

BM: Bone marrow; PB: Peripheral blood; BMMC: Bone marrow mononuclear cells; PBMC: Peripheral blood mononuclear cells; PMA: Phorbol 12-myristate 13-acetate; CMV: Cytomegalovirus; CDR3: Complementary determining region 3; RFU: Relative fluorescence unit; APC: Allophycocyanin; MACS: Magnetic activated cell sorting

## Competing interests

None of the authors has competing interests.

## Authors' contributions

TP, BW and KLR performed experiments and analyzed the data. RR, JL, RG and MK provided bone marrow samples. DHB provided the iliac crest-derived samples as well as some of his staining results. DHB and BGL designed the study protocol. TP, BW and BGL wrote the manuscript. All authors read and approved the final manuscript.
